# Impact of digitalization on clean governance: An analysis of China’s experience of 31 provinces from 2019 to 2021

**DOI:** 10.3389/fpsyg.2022.947388

**Published:** 2022-07-29

**Authors:** Du Xiaoyan, Majid Ali, Xu Le, Wu Qian, Gao Xuelian

**Affiliations:** School of Marxism, Xi'an Jiaotong University, Xi'an, China

**Keywords:** TOE framework, open data, digitalization, fsQCA analysis, clean government

## Abstract

The goal of deepening institutional reforms was to bring transparency and accountability, address corruption, and establish a clean government (CG) in China. The first step toward this transparency is considered to be the free development and transmission of Open Data (OD). In this regard, China has set up open data centers in provincial governments. Considering that OD can have an impact on CG and bring new ideas for CG construction, ODs of 31 provincial governments have been analyzed through fsQCA3.0 to test these assumptions. To see how much it can contribute to the development of the Technology Organization Environment Framework (TOE). To this end, between 2019 and 2021, 31 provincial government data have been clustered into low, medium, and high corruption case enrollment areas to determine the impact of OD. The study mentioned that improvements in ODs in 31 provinces could strengthen cooperation with the disciplinary inspection department in the fight against corruption. The study, on the other hand, made two assumptions that environmental barriers and internal pressures could affect data’s reliability.

## Introduction

Establishing a clean and effective government has always been an important goal that governments around the world, including China, are pursuing. Since the 18th Communist Party of China (CPC) National Congress and Chinese local governments have actively used open data in the fight against corruption and pushing for a clean government. Experimental evidence from China and around the world shows that open data can not only improve the efficiency of government management but also improves the credibility of the government (Ricardo and Marijn, 2020). Open Data (OD) is based on Information Communication Technology (ICT), the latter produces data from various governmental and nongovernmental platforms, develops its use, circulate, transform, and implement the use of it for effective e-governance to empower CG (Chaobing and Wenqiang, 2020).

Currently, researchers are more interested in knowing the relationship between governance and open data and pay less attention to the reliability of open data and its impact on government decisions. Open Data (OD) has an impact on the governments of the Netherlands, Canada, France, and Singapore, because they developed open government data policies to curb corruption, which has a positive impact on CG (Mei and Wei, 2019).

In recent years, Chinese local governments started following the OD experiment to curb corruption and malpractices, with the help of the Department of Discipline and Inspection. It also launched surveillance for information-based anti-corruption campaigns jointly with the help of relevant departments. Having set up big data laboratories and big data platforms, the agencies gained a lot of practical experience in investigating the case during the patrol inspections and found some lapses (Qiang et al., 2019; Xiangbo and Lin, 2022). These experiments showed that open data can effectively improve the government’s credibility, which requires academician’s further investigation. During the 14th 5-Year-Plan period in China, big data centers have been built and put into use. The government implemented the “leaving the traces” methodology for catching the cases of malpractices in office or during their official term. Most of the time, the government is generating quantitative data for administrative examination and cross-checking with the pre-approvals to develop materials about procurement procedures and public service procurements methodology. The effective use of open data can efficiently contribute to the improvement of the reliability of the governments. Therefore, in the current formation of clean government initiatives, our study should not only focus on combating corrupt maneuvers but also on the initiatives of open data for systematic governance and sustainable prevention of corruption.

### Research questions

How open data can affect clean governance of government and how to promote the development of clean governments with open data are the key questions of this paper.

## Theoretical background

In the information age, governments have a lot of reliable data for important governmental decisions (Dongfang, 2021). To address effective governance during crises, Western societies have come up with a “digital governance theory” as a solution. For the first time, in his book Digital Era Governance (2006), Patrick Dunlevy systematically articulated the concept of digital governance. Corporations, states, and e-governments pointed out that the digitalization of governance has changed the traditional organizational structure of government, and digital technology has become a part of the whole process of government governance (Dunleavy, 2006). In the COVID-19 hunted world, schools are delivering online classes, business meetings, and politicians’ meetings are virtualized, officials can work from their homes, and even technologies are suggested for construction works like Belt and Road Initiative (Zhong et al., 2022). In China, Zhu Qianwei scholar was the first to introduce the theory of digital governance. He introduced the theory in detail in his book “Public Administration Theory” published in 2008 and believed that it was a new transcendence of the current government’s holistic governance approach (Qianwei, 2008). Digital governance theory emphasizes the concept of development through data sharing and scientific decision-making. The process of redesigning data enables government decisions to eliminate discrepancies between traditional and modern e-led systems that are more accurate, efficient, and collaborative than traditional (Feiwei and Jingwen, 2021).

This paper takes digitalization theory as the theoretical basis of research. One of the important aspects of digital governance is the production of open data and the formation of massive standardized data for policy development and implementation. The government’s open data are the result of innovation, legalization, and technology diffusion along with policy implementation (Chunkui et al., 2021). China ranks 45th out of 193 countries in the world in the 2020 United Nations e-Governance Survey report with a score of 0.7948; 162 countries have an online open platform for official data and 59 have developed open government data policies (Chunkui et al., 2021). Digitalization may be seen as an inevitable outcome of modern governance style in the age of information for speedy development. The Chinese government fully recognizes the need for digitalization, its importance, and its implementation with a top to bottom approach, that is why in 2018, the State Council of China promulgated The Deepening the implementation of the internet Plus Government Services to promote the implementation of “one network, one door, like 国家政务服务平台用户指引(Guójiā zhèngwù fúwù píngtái yònghù zhǐyǐn)” in other words, a guide to National Government Platform (GJZWFW, 2020). Digitization is needed at all levels of government ranking so that access to it can be continued and institutional barriers can be broken and a unified national network management services system can be gradually established (General Office of the State Council, 2018).

In the year 2018, the China National Development and Reform Commission (NDRC), the Internet information office, and the Ministry of Industry and Information Technology of China jointly issued the pilot work plan for the opening of public information centers, requiring the pilot opening of Government Public Information Data Center in Beijing, Shanghai, Zhejiang, Fujian, and Guizhou and formed a joint national open data platform clarifying the scope of opening and data utilization (The Central Internet Information Office, the Development and Reform Commission, and the Ministry of Industry and Information Technology jointly carried out the pilot work of open public information resources, 2018). In 2019, the regulations of the People’s Republic of China on government information disclosure were promulgated and implemented; requiring local governments at all levels to follow the principle of openness as standardization and released the relevant government data and information in a timely manner with accuracy and authenticity. In January 2022, the Chinese State Council again requested The Data Economy Development Plan 14th 5-Year-plan to increase the degree of openness of local government data and enhance the efficiency of Internet-based government and services (“Fourteenth Five-Year” digital economic development plan, 2022). China is currently working on an open data era to balance the local governments. As of October 2020, 142 provisional and municipal governments across China had set up open data platforms, including Zhejiang, Shanghai, Beijing, and Guizhou (Feng and Chunxia, 2022).

Corruption is known as “political cancer” and Xi Jinping called corruption a tree worm that eats trees from within (Jinping, 2017, p.181), as characterized by concealing crimes, case complexity, and group involvement. Building a clean government is an important goal of the Chinese government. Since the 18th National Congress of the Communist Party of China (CPC), an anti-corruption campaign has been launched to curb corruption. Governments at all levels have taken the initiative and tried to stick with three principles: digitalization of data, acceleration of clean government, and enhancement of government credibility. The investigation and prevention of corrupt practices need open and reliable data. The research finds that opening government data can have a positive effect on CG (Vrushi and Hodess, 2017; Shaoyou et al., 2018).

The McKinsey Global Institute argues that transparency and accountability in government can be enhanced by the digitalization of governments, which in return improves the quality of government services (Manyika et al., 2013). That is why our previous study clubbed CG and digitalization of Beijing’s motivation as striving toward transparency (Ali et al., 2022). Through empirical analysis, Worthy (2015) found that the availability of government data promotes oversight and decreases the probability of corruption. In the next 5 years of China’s government reform, the important goal is to expand the orderly opening of basic public information data; the development of national interconnected data sharing *via* platforms is to foster a sound political environment in which people by any means are not allowed to corrupt practices (Proposal of the Central Committee of the Communist Party of China on formulating the 14th Five-Year Plan for National Economic and Social Development and the long-term Goals for 2035, 2020). To promote open data in the fight against corruption and to scientifically verify the sources of corruption, open government data must be available. Clean governance requires deep reforms in open data, and promoting clean government requires further refinement of its relationship with open government data in the fight against corruption.

### Research hypothesis

In the development of clean government, open data play an important role as a powerful tool for discovery, investigation, and prevention. Peisakhin and Pinto (2010) analyzed the relationship between the impact of openness and honesty of official data in 14 countries and found a significant positive relationship between the two. This shows that open data can play an extraordinary role in controlling and governing corruption (Peisakhin and Pinto, 2010). Transparency International highlighted the importance and effect of open data in the containment of corruption and enhancement of efficient governance; it analyzed data from 2014 to 2015, in which 95% of cases of corruption revealed that open data have a direct impact on case investigations (Roger and Tim, 2016).

This is corroborated by research of Vrushi and Hodess (2017), who found an 80% correlation between the Corruption Perceptions Index and the Open Data Barometer (Vrushi and Hodess, 2017). Rajshree and Srivastava (2017) believe that open data can improve the government’s transparency and promote accountability by the government, thus reducing the chances of corruption (Rajshree and Srivastava, 2017). Recently, the acceleration of the digitalization of government and the strong implementation of the open data policy of the Chinese government have shown an applauding growth in performance. From January to September 2021, discipline inspections and supervision entities across the country handled 1,364,000 complaints and filed 470,000 cases, among which the government’s open data played a positive role in the identification and investigation of cases of corruption (Yadong et al., 2022). Based on the above research, this paper proposes a research hypothesis: that there is a correlation between open data and clean government, the higher the degree of data is open, the higher the government is transparent and reliable.

### Theoretical framework application

In recent years, it is noted that some scholars are using the theoretical framework of “technology organization environment” (TOE) to analyze government’s open data. Zhiwei and Yan (2020) used the TOE framework to analyze the utility of the provincial government’s open data platforms in 13 provinces and divided it into three different types: internal and external relationship of provinces, technology and organization relationship, and organizational environment (Zhiwei and Yan, 2020). Shuyan and Hupa (2021) compared the performance of 20 provincial government’s Open Data systems from eight different aspects under the TOE framework and proposed four ways of high-level generation of open data and improvement approaches (Shuyan and Hupa, 2021). Jiangping et al. (2022) conducted a configuration analysis on the factors influencing the development of big data in government affairs at 31 provincial levels based on the TOE framework, classified seven different types, and proposed three development modals. These studies focus on the size of government open data and ignored the discussion and evaluation of synergistic effects of multiple factors brought by government OD (Rihoux and Charles, 2017). In this paper, based on the TOE framework, the clean government (CG) is set as a dependent variable, and the following mathematical analysis model is established to further analyze the relationship between the government’s effectiveness with open data.


$$\begin{array}{l} f\left(Y_{L}\right)=r\times T+t\times E+p\times O\\ \begin{array}{l} =r\times \left(\overset{3}{\underset{i=1}{\sum }}s_{1i}\times Ta_{i}+\overset{5}{\underset{i=1}{\sum }}s_{2i}\times Tb_{i}+\overset{4}{\underset{i=1}{\sum }}s_{3i}\times Tc_{i}+\overset{4}{\underset{i=1}{\sum }}s_{4i}\times Td_{i}\right)\\ +t\times \left(j\times E_{1}+k\times E_{2}\right)+p\times O \end{array}\\ \begin{array}{l} =r\times \left(\overset{3}{\underset{i=1}{\sum }}s_{1i}\times \frac{r_{1}}{r}+\overset{5}{\underset{i=1}{\sum }}s_{2i}\times \frac{r_{2}}{r}+\overset{4}{\underset{i=1}{\sum }}s_{3i}\times \frac{r_{3}}{r}+\overset{4}{\underset{i=1}{\sum }}s_{4i}\times \frac{r_{4}}{r}\right)\\ +t\times \left(\frac{t_{1}}{t}\times E_{1}+\frac{t_{2}}{t}\times E_{2}\right)+p\times O \end{array}\\ \begin{array}{l} =r\times 1\overset{3}{\underset{i=1}{\sum }}s_{1i}+r_{2}\times \overset{5}{\underset{i=1}{\sum }}s_{2i}+r_{3}\times \overset{4}{\underset{i=1}{\sum }}s_{3i}+r_{4}\times \overset{4}{\underset{i=1}{\sum }}s_{4i}\\ +t_{1}\times E_{1}+t_{2}\times E_{2}+p\times O \end{array} \end{array}$$

Among them, $Y_{L}$ on behalf of the provincial government, clean Government, T on behalf of the provincial government, open data, T is decided jointly by $\left(\overset{3}{\underset{i=1}{\sum }}s_{1i}\times Ta_{i}+\overset{5}{\underset{i=1}{\sum }}s_{2i}\times Tb_{i}+\overset{4}{\underset{i=1}{\sum }}s_{3i}\times Tc_{i}+\overset{4}{\underset{i=1}{\sum }}s_{4i}\times Td_{i}\right)$ , in which $\overset{3}{\underset{i=1}{\sum }}s_{1i}$ said policy Ensuring, $\overset{5}{\underset{i=1}{\sum }}s_{2i}$ said open data Online platform’s Availability, $\overset{4}{\underset{i=1}{\sum }}s_{3i}$ said open data technological Embeddedness, $\overset{4}{\underset{i=1}{\sum }}s_{4i}$ said Open Data usefulness, O on behalf of the organization Size, and E on behalf of the environmental constraints. The constraint condition is composed of $E_{1}$ (representing the pressure within the province) and $E_{2}$ (representing the pressure outside the province), and the other parameters in the formula represent different influences coefficients.

#### The provincial government’s clean governance ( $Y_{L}$ )

This is an established claim of the study that if there are few complaints or petitions against any government’s authority or institution or allegations of corruption, it indicates the effectiveness of governance, whereas on the other hand, if the cases are numerous against any institution or individual and there are more applications or complaints registered against it shows the malpractices. Less reported corruption is a sign of a Clean Government. This study found that out of 31 provinces, 18 are reported as less corrupt, based on less reported criteria, which are on the other hand cleaner (Table 1).

**Table 1:** Registered cases-based table (2019-2021).

S.No	Provinces	PPE	POPA	PTLE	PODU	POL	CPIn	CPOn	CGE	RC
1	Fujian	7.46	7.87	17.47	6.1	3383.38	0.67	3.67	5.772200772	299
2	Beijing	4.24	4.58	21.51	10.7	5932.31	2	2	8.211586902	326
3	Tianjing	6.13	5.4	21.73	7	2141.04	2	2	14.68208092	254
4	Jiangxi	7.3	4.5	13.56	6.2	2812.25	0	2.5	4.308681672	268
5	Qinghai	0.8	0	0	4	330.76	0	1	10.92105263	166
6	Shanghai	12.39	15.12	26.73	16.5	7771.8	5	3.5	11.9	238
7	Guangxi	9.87	8.69	21.23	3.6	1800.12	0.86	1.2	7.567567568	448
8	Hainan	4.63	4.62	3.61	6.2	921.16	0	4	15.03355705	224
9	Guizhou	12.07	11.95	11.71	15.4	1969.51	0.33	1.6	6.810810811	504
10	Hainan	1.5	2.8	2.36	0	2775.27	0	1.25	5.658536585	348
11	Xinjiang	0.8	0.64	0.69	0	1618.6	0	0.33	4.670658683	390
12	Ningxia	2.1	7.65	13.49	0.5	460.01	0	0.67	5.897435897	69
13	Tibet	1.2	0.71	0	0	215.59	0	0.75	14.66216216	217
14	Gansu	0.7	3.43	3.35	2	1001.83	0	1	5.899419729	305
15	Chongqing	5.03	4.78	11.93	5.6	2285.45	0	1.8	7.531806616	296
16	Anhui	3.43	5.1	1.99	2	3498.19	0	2.83	8.523489933	508
17	Jilin	3.14	0	0	0	1143.97	0	0	10.75520833	413
18	Hebei	0.7	2.25	3.53	4	4167.58	0	1.57	5.682051282	554

#### Provincial level data openness ( T )

The open data can be subdivided into four conditional variables: “policy Ensuring (PE),” “Online platform availability (OPA),” “Technological Embeddedness (TLE),” and “Open data usefulness (ODU).” Among them, the “PE” consists of three sub-indicators, namely, open data regulations and policies, organizational implementation drive, and standardization of the specific formulation, which are used to measure the infrastructure development and improvement’s growth of local government’s open data (Xinping et al., 2019). “Online Platform Availability (OPA)” consists of five sub-indicators, including platform’s data development, platform’s data acquisition, platform’s data exchange, platform’s interactive feedback, and user’s experiences, which are used to measure the function of Open data government [5]. The “technological Embeddedness (TLE)” consists of four sub-indicators, namely, data quantity, data quality, data specification, and scope, which are used to measure the technical stage and open data’s scope of government data. “Open Data Usefulness (ODU)” consists of four sub-indicators, namely, increase of data utilization demand, quantity of effective results, quality of effective results, and multiplicity of data, which are used to measure the effective utilization of government open data; the below Table 2 provides detailed information about provincial level data openness.

**Table 2:** Provincial data openness score (2019-2021).

S.No	Provinces	PPE	POPA	PTLE	PODU	POL	CPIn	CPOn	CGE	RC
1	Zhejiang	15.26	13.88	30.45	17.1	8262.57	2.27	3	9.396863691	779
2	Shandong	11.18	13.38	24.24	17.3	7284.45	3.06	2	7.076923077	966
3	Guangdong	7.54	10.84	25.78	5.1	14103.43	0.67	2.5	7.851405622	1,173
4	Fujian	7.46	7.87	17.47	6.1	3383.38	0.67	3.67	5.772200772	299
5	Beijing	4.24	4.58	21.51	10.7	5932.31	2	2	8.211586902	326
6	Tianjing	6.13	5.4	21.73	7	2141.04	2	2	14.68208092	254
7	Sichuan	6.1	9.96	17.19	11	4773.27	0.44	0.71	8.896860987	992
8	Jiangxi	7.3	4.5	13.56	6.2	2812.25	0	2.5	4.308681672	268
9	Henan	1.5	3.84	12.49	0.5	4347.38	0	1.83	6.501650165	788
10	Hubei	5.04	1.68	0.19	0	3283.3	0.23	1.5	13.33333333	1,016
11	Qinghai	0.8	0	0	4	330.76	0	1	10.92105263	166
12	Shanghai	12.39	15.12	26.73	16.5	7771.8	5	3.5	11.9	238
13	Guangxi	9.87	8.69	21.23	3.6	1800.12	0.86	1.2	7.567567568	448
14	Hainan	4.63	4.62	3.61	6.2	921.16	0	4	15.03355705	224
15	Jiangsu	1.4	1.92	1.32	6.8	10015.16	0.23	4	10.56179775	940
16	Guizhou	12.07	11.95	11.71	15.4	1969.51	0.33	1.6	6.810810811	504
17	Shaanxi	1.5	2.8	2.36	0	2775.27	0	1.25	5.658536585	348
18	Xinjiang	0.8	0.64	0.69	0	1618.6	0	0.33	4.670658683	390
19	Ningxia	2.1	7.65	13.49	0.5	460.01	0	0.67	5.897435897	69
20	Tibet	1.2	0.71	0	0	215.59	0	0.75	14.66216216	217
21	Yunnan	1.2	0	0	0	2278.24	0	2.25	13.18318318	878
22	Gansu	0.7	3.43	3.35	2	1001.83	0	1	5.899419729	305
23	Inner Mongolia	2.5	0.58	0	0	2349.94	0	0.875	12.93814433	753
24	Chongqing	5.03	4.78	11.93	5.6	2285.45	0	1.8	7.531806616	296
25	Shanxi	4.44	1.09	0	0	2834.61	0	1	12.14733542	775
26	Anhui	3.43	5.1	1.99	2	3498.19	0	2.83	8.523489933	508
27	Heilongjiang	1.2	0.65	2.1	0	1300.5	0.17	0	14.8427673	708
28	Jilin	3.14	0	0	0	1143.97	0	0	10.75520833	413
29	Liaoning	1.4	0	0	0	2764.71	0	0.67	9.631811487	654
30	Hunan	0.8	3.23	0.14	1.5	3250.69	0	2.17	9.764837626	872
31	Hebei	0.7	2.25	3.53	4	4167.58	0	1.57	5.682051282	554

Clean Government Effect means: Number of corruption cases per 10,000 people in a province. Provincial Policy Ensuring (PPE); Provincial Online Platform Availability (POPA); Provincial Technological Embeddedness (PTLE); Provincial Open Data Usefulness (PODU); Provincial Organization Level (POL); Competitive pressure to open data of different cities in the same province (CPIn); Competitive pressures brought by the development of open data in other provinces (CPOn); Clean Government Effect (CGE); Registered Cases (RC).

#### Organizational size ( O )

The organizational efficiency is illustrated by the organization’s financial capacity, which is also a conditional variable. The financial capacities of local governments are directly affecting the internal efficiency of organizations. In empirical studies, the financial capacity is generally expressed by the government’s annual financial revenue, and it is believed that this index can be used to measure the worth of a government organization. For example, Zhiwei and Yan (2020) used this indicator to measure the organizational stature of local governments in his paper “Configuration Analysis of Utilization Level of Government Data Open Platform under THE TOE Framework” (Zhiwei and Yan, 2020). This paper also selects the fiscal revenue of provincial governments to represent the organizational level (Table 2).

#### Environmental constraints ( E )

This variable is composed of two sub-indicators, “pressure in the province CPIn( $E_{1}$ )” and “pressure out of the province CPOn ( $E_{2}$ ).” The government of China accelerated the E-governance initiatives of provincial governments by establishing a common platform. This common platform is not only working as a competition or accelerator among government sister organizations but also working as a pressure developer. They may also come in competition with the international community for service delivery, which is identified as external pressure according to pressure categorization. Internal pressure is interprovincial competition and external pressure is competition with internal community (Huang and Xuezhi, 2018).

**Figure 1:**
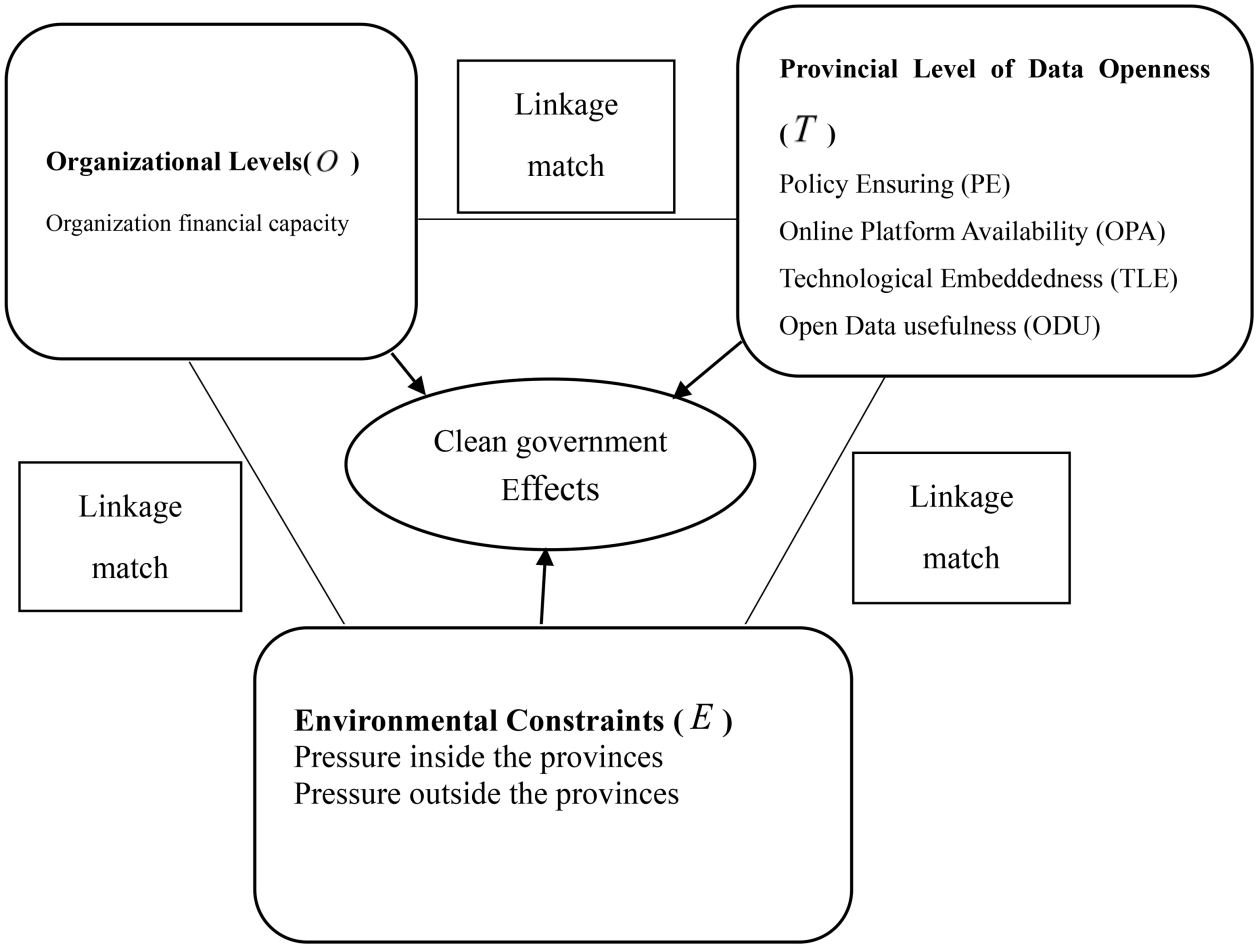
The government integrity conceptual model.

Based on the TOE framework, a conceptual model of clean government effect with influencing factors developed is shown in Figure 1.

## Variable selection and study outline

### Methodology

In traditional quantitative analysis, too much attention is paid to the influence of a specific variable and its results, but this influence is often not as simple as it is taken for its relationship. If the result is completely attributed to a single factor effect, then the result is bound to have some inclinations toward variables or toward case studies, so it is necessary to consider the joint effects of variables and case studies. Therefore, fuzzy set qualitative comparative analysis (fsQCA) is selected for data analysis in this paper. fsQCA is a comparative analysis method based on Boolean algebra, which treats each case as a “part” of a series of conditional variables. This paper tries to explore the effect of open data on governments’ righteousness, used by some of the variables of the sample analyzed, and the two indicators to verify the independent variables of the study are open data logical conditions established to find the relationship between the government open data and its impact on clean governance.

As a country with a special political system, China not only emphasizes the high Command of the Party Central Committee and the State Council but also grants 31 provincial governments certain policy-making powers such as open data. Judging from the actual development situation in China, local governments have formulated different open data policies according to local special conditions, carried out many autonomous attempts to use open data to control corruption, and formed different models of open data and clean government effects. However, many Chinese scholars analyze the policies and effects of government open data at the national level, ignoring the particularity of local governments. At present, there are just a few articles that do empirical research on government open data from the local level, and only a few articles focus on the relationship between open data and clean local government. This paper wants to analyze the deep relationship between open data policy and the clean local governments, and use the fsQCA to divide the local governments using open data level to anti-corruption into different types, find the core variables, and then give some suggestions to improve clean government effect by optimizing open data.

The fsQCA is a methodology for using dig deeper into data to reveal minute details about the complexity of the relationship between open data and clean government at the provincial level. fsQCA methods are compatible with data asymmetry, potential interdependencies of variables, identify asymmetric data relationships, and reveal multiple equivalence paths to the same outcome. fsQCA examines the relationship between an antecedent variable within a case and analyses the relationship between the dependent variable and a specific combination of conditions. It finds common configurations of multiple cases, and these different common configurations constitute specific pathways for specific outcomes. Thus, fsQCA complements traditional symmetric approaches by adding a more nuanced understanding of entrepreneurial phenomena and providing an empirical basis for analysis, that is, it can disclose astonishing empirical findings that inspire new theory-building for trying in another direction.

Fuzzy set qualitative comparative analysis 3.0 software is statistical analysis software, which was used in this paper to analyze the relationship between open data and clean government’s effect on China’s 31 provinces.

### Data sources and standardization

#### Data sources

The article mainly solves five problems. First, using the data published by the authoritative statistical report “China Local Government Data Openness Report” from 2019 to 2021, analyzing the data openness level of 31 provinces across the country. Second, building the analysis model of open data and clean local government based on the TOE framework. Third, the fsQCA method is used to analyze the relationship between open data and the clean local government effect. Fourth, using panel data from 2019 to 2021, this paper found that the core conditional variables that affect the clean local government effect are open data’s policy guarantee index which is called “Policy Ensuring (PE),” open data’s platform construction index, which is called “Online Platform Availability (OPA),” and open data’s technology level index which is called “Technological Embeddedness TLE.” Fifth, the fsQCA 3.0 software is used to analyze the different types of the relationship between open data and clean local government effect. According to the analysis results, there are four models, such as open data policy guarantee and government organization-driven level model, open data technical level and government organization-driven model, open data platform construction and government competition environment model, and government organization-driven level and open data utilization transformation model. Finally, the article puts forward some suggestions to enhance the local government open data level to enhance the cleanness of local government effect.

The data in this paper are mainly from the websites of the National Bureau of Statistics, the provincial bureau of Statistics, the Supreme People’s Procuratorate, and the Fudan University’s (2021) Report on The Opening of Chinese Local Government Data (see Table 2). The data are selected from 2019 to 2021. The annual number of registered discrepancies in China’s 31 provincial-level administrative regions (excluding Hong Kong, Macao and Taiwan) is based on data from the websites of the Supreme People’s Procuratorate and the Supreme People’s Court. The provincial open data have been categorized into four indicators: “Policy Ensuring (PE),” “Online Platform availability (OPA’s),” “Technological Embeddedness (TLE),” and “Open Data Usefulness (ODU).” The data are all from the reports on The Opening of Chinese Local Government Data published by the Digital and Mobile Governance Laboratory of Fudan University.

The report, jointly launched by Fudan University and the Digital China Research Institute of the State Information Center, has become an authoritative report on monitoring the level of data openness of Local governments in China (“China Local Government Data OpenNess Report”). The data of government organization level come from the annual financial revenue of the governments of provinces (autonomous regions and municipalities directly under the Central Government) in the China Statistical Yearbook. The environmental constraint conditions are composed of “provincial internal pressure” and “neighboring province pressure,” and the data come from China Local Government Data Opening Report. The specific situation of each variable is shown in Table 3.

**Table 3:** Measurement indicators and data sources.

Variable types	Variable name	Measurement	Sources
Result	Clean Government effect	Number of corruption cases registered per 10000 people in the province	Websites of the Supreme Procuratorate and the Supreme People’s Court from 2019 to 2021
Open data status	Policy ensuring (PE)	Open data regulations and policies of provincial governments	China Local Government Data Opening Report(2019–2021)
		Organizations and implementation of open data by provincial governments	
		Development of open data standards and specifications by provincial government	
	Online platform availability (OPA’s)	Development of provincial government open data platforms	
		Data acquisition from open data platforms of provincial government	
		Exchange of data among provincial governments *via* open data platform	
		Provincial government open data platform’s feedback	
		Users experiences	
	Technological embeddedness (TLE)	The provincial governments releasing amount of data	
		Quality of Provincial governments opens data	
		Detailed information about Provincial governments open data	
		Scope of provincial government’s open data the scope	
	Open data usefulness (ODU)	The provincial government opens data utilization	
		Open data’s number of results by the provincial government	
		Affectivity results	
		Opening of divers data for utilization	
Organizational strength	Fiscal capacity	The annual financial revenue of provincial government	China Statistical Yearbook (2019–2021)
Environment constraints	Pressure to the province	Average value of forests index in prefecture-level cities of this province	China Local Government Data Opening Report(2019–2021)
	Neighboring pressure	Average forest index of neighboring province	

#### Standardization of results

When fsQCA3.0 software is used for analysis, each conditional variable and result is regarded as an independent set, and each case has its relative score in these sets, which requires a data standardization process. In this paper, the direct standardization method was used to convert the data into relative scores of fuzzy sets (for Further Understanding consult; Castelló-Sirvent and Pinazo-Dallenbach, 2021). The full standard was set at 0.95, the intersection calibration standard at 0.5, and the complete non-membership calibration standard at 0.05. Table 4 shows the calibrated data of each condition variable and result variable, and the results show that the data can be used for configuration analysis.

**Table 4:** Data calibration and standardization (2019-2020).

Variable	Conditions and results indicator	Calibration		
		Full membership calibration complete	Intersection maximum ambiguity	Non-membership calibration
Clean Government Effect	Number of corruption cases per 10000 people in a province	954.5	525	84.5
Open data level	PE	13.215	2	0
	OPA	15.8	3.28	0
	TLE	23.93	8.14	0
	ODU	11.525	1	0
Organizational levels	Fiscal capacity	8153.615	2296.57	360.785
Environmental constraints	Internal pressure of province	3	0	0
	Eternal pressure of province	3.165	1.5	0

## Results analysis

### Conditional analysis

We need to check the necessity of each variable before conducting a configuration analysis. In fsQCA, the precondition for taking a case study as a necessary condition for the result is that its consistency alignment should reach 0.9. The fsQCA3.0 software analyzed the necessity of each prerequisite condition, and the results are shown in Table 5. It can be seen from Table 3 that the consistency level of all the conditional variables we tested is less than 0.9, indicating that these variables cannot independently constitute an indispensable environment for influencing the government clean project effect, because the clean government can be affected by multiple factors. Therefore, it is necessary to further analyze the synergistic influence and relationship between all variables of the provincial government’s clean effects from three aspects, the level of openness of data, the level of organization, and the level of environmental constraints.

**Table 5:** Necessary conditions analysis (2019-2021).

Condition variables	Consistency interpretation of individual variable	Results of variables
PE: production of government open data	0.527359	0.580096
Non-policy protection: Non-productive	0.705285	0.606273
OPA	0.448454	0.482001
Non-platform construction index	0.778841	0.681998
TLE	0.425016	0.424746
Non-technical level	0.736097	0.686808
ODU	0.472494	0.562401
Non-utilization	0.742100	0.602225
Financial capacity	0.562617	0.592469
Non-financial capacity	0.707397	0.630037
Pressure to the province (competition within province over digitalization of different cities)	0.750630	0.610878
Non-provincial pressure	0.578213	0.685389
Neighboring pressure	0.600301	0.603934
Non-neighbor pressure	0.657746	0.609919

### Conditional configuration analysis

Fiss, emphasized that “organizations cannot be understood in an isolated analysis, because organizations are interconnected clusters of practices” (Fiss, 2007). When conducting configuration analysis, we usually adopt the holistic idea and pay attention to the combined effect of condition variables rather than to the analysis of single variables conditions. In conditional configuration analysis, the screening of PRI consistency and full consistency is particularly important. The full name of PRI is “Proportion Reduction Inconsistency,” and the higher PRI consistency, the less possibility of “same cause and different results.” The PRI frequency threshold should be determined according to the sample size, and the consistency of PRI should be no less than 0.5. Sufficient consistency refers to the proportion of the membership set of the result variables and as a subset of the conditional variable to the membership set of the result variable and the level of sufficient consistency should not be less than 0.85. The sufficient consistency threshold adopted in this paper was 0.85. Due to the small sample size, the frequency threshold was selected as 1, and the data with PRI consistency less than 0.5 were screened.

This paper uses fsQCA3.0 software for conditional configuration analysis, and Table 6 shows the analysis results of different configurations. The results show that there are six condition combinations and four configurations between the government integrity effect and the level of open data. The total consistency of variables is 0.6013, indicating that 60.1% of the four configurations have a good honesty effect on provincial governments, and the total coverage is 0.8879, indicating that the combination of six conditions can cover 88.79% of explanatory variables, which also indicates that the conditional variables selected in this paper have a strong explanatory power on the honesty effect of provincial governments, see for details Castelló-Sirvent and Pinazo-Dallenbach (2021). The overall consistency and middle coverage of variables are both higher than the critical value, indicating the validity of this research analysis, and also proving the validity of our research hypothesis that “the degree of government data opening is positively correlated with the honesty effect to a certain extent.” Based on the analysis results, we divided the conditional variable configuration of provincial government integrity effect and open data level into four models.

**Table 6:** Conditional configuration analysis of honesty effect in the provincial government (2019-2020).

Conditions or pathways	Configuration Model 1	Configuration 2	Configuration 3	Configuration 4
CG Effect	⊙	⊙	⊙	⊙
PE	●	⊙	⊙	⊗
OPA	⊙	⊘	●	⊙
TLE	⊙	●	⊙	⊙
ODU	⊘		⊗	⊙
Fiscal capacity	⊙	⊙	⊘	●
Pressure to the province	⊗	⊙	⊙	⊙
Neighboring pressure		⊘	⊗	⊘
Raw coverage	0.389733	0.300241	0.177483	0.217207
Unique coverage	0.0894918	0.0273955	0.0183288	0.0658796
Consistency	0.943173	0.814564	0.760803	0.735393
Directional Consistency	0.601337			
Expectations	0.887939			

⊙: There is only an intermediate solution, and the condition is “yes”; ⊘: There is only an intermediate solution, and the condition is “no”; ●: There exist both simple and intermediate solutions. The condition is “yes”; ⊗: There exist both simple and intermediate solutions. The condition is “no”; and empty: Independent of this condition.

The first approach is the policy guarantee—organizational ambitious mode, corresponding to configuration 1. In this modal, the PE is the core conditional variable, and the government organization size OPA, TLE, and provincial pressure are the Minimum conditional variables, which also have a direct impact on the clean government effect. The original coverage of this model is 0.389, which can explain 38.9% of provincial government cases, and the full consistency is 0.943, indicating that 94.3% of cases can be explained by this model, which also indicates that this model has universal applicability.

The second modal is TLE, corresponding to configuration 2. In this mode, the open data technology level index is the core conditional variable, while the government organization size, PE, and provincial pressure are the minimum conditional variables, which will have a direct impact on the Clean government (CG) effect. The original projection of the model is 0.30, which could explain 30% of the registered cases, and the full consistency was 0.814, which could explain 81.4% of the cases dealt.

**Figure 2:**
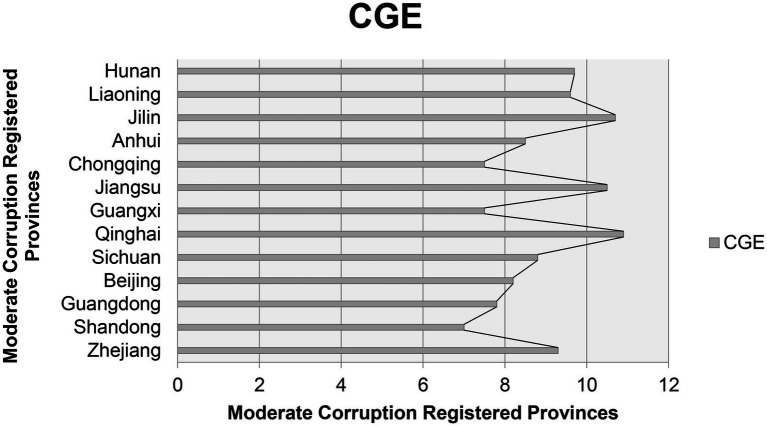
Moderate corruption registered cases.

**Figure 3:**
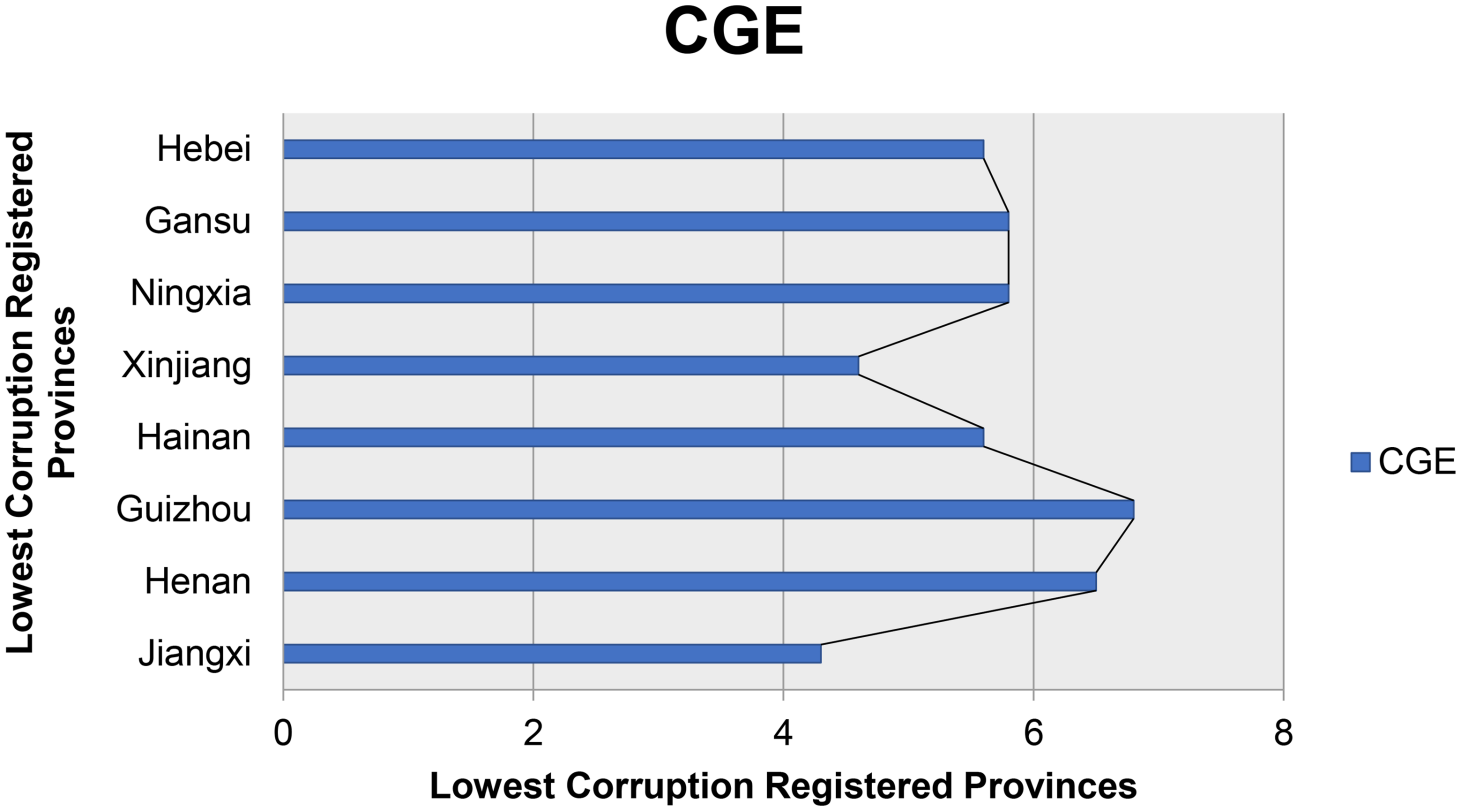
Lowest corruption registered provinces.

**Figure 4:**
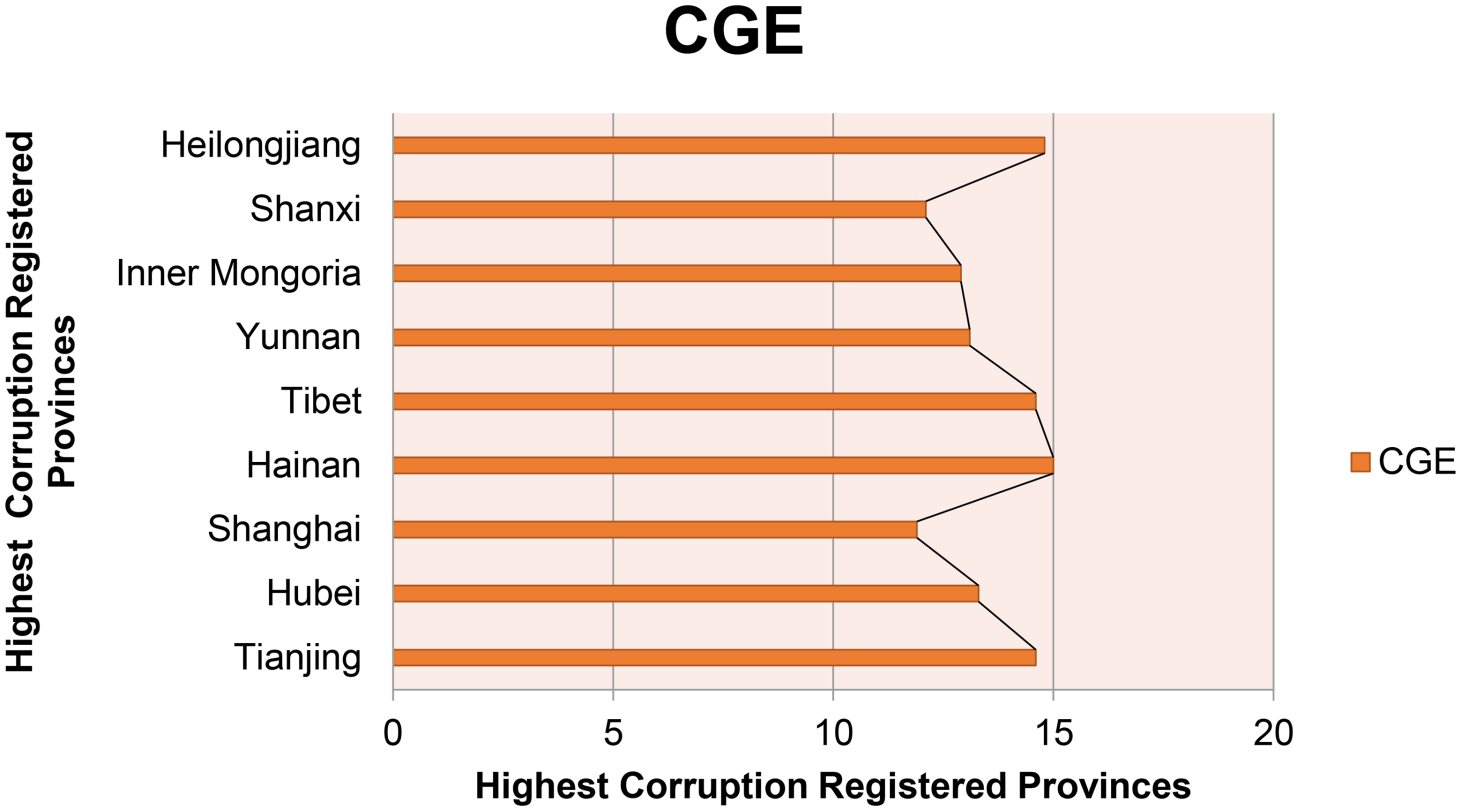
High corruption registered provinces.

The third modal OPA is driven by competition among provinces, corresponding to configuration 3. In this mode, the open data platform is the core conditional variable, and the provincial pressure, PE, and TLE are the minimum conditional variables, which will have a direct impact on the CG. The original coverage of this model was projected at 0.177, which can be explained in 17.7% of cases, and the full consistency is 0.761, which is explained in 76.1% of cases. The number of growth of registered cases dealt with by an individual province compared with Moderate (Figure 2) and lowest registered cases (Figure 3) of provinces is shown in Figures 2, 4.

The fourth modal is TLE, corresponding to configuration 4. The core conditional variable and transformation index, OPA, technology level (TL)^1^ index, and provincial pressure are the conditional variables, which will have a direct impact on the CG. The original coverage of the model was projected at 0.217, which could explain 21.7% of the cases, and the full consistency was at 0.735, which could explain 73.5% of the cases.

The above four configuration modes indicate the level of openness of data, which have a strong correlation with the clean government effect, and the PE, OPA, and TLE are all four conditional variables that have effects on clean government. Of course, the size of a governmental organization represented by its financial capacity is also a conditional variable, indicating that the level of a governmental organization also has an important impact on the Clean Government. Provincial pressure, such as environmental constraints, is a marginal condition in the second and third models, which has a direct impact on the Clean Government. This indicates that the driving force of open government data is more from the competition within different prefecture-level governments within the province.

### Robustness test

In configuration analysis, it is necessary to conduct further robustness tests to avoid the possible and noticeable deviations caused by limitations value and consistency thresholds due to certain possibilities in the selection of values. In this paper, robustness analysis was conducted after appropriately increasing the values of intersection points, and it was found that the four configurations shown in Table 4 still stand true. Then, the consistency threshold was adjusted to 0.8, and the solution results were also highly similar in consistency and coverage (the consistency of the solution was 0.598526 and the coverage of the solution was 0.853105). According to the above results, we can justify four Configurational results (Table 6) impact on Clean government.

Open data improve government transparency and facilitate economic development and innovation. In recent years, many countries started open data initiatives. In recent years, open government data have been rapidly promoted in many countries and have become a hot topic and research focus in the field of government informatization. In May 2009, the US federal government data open platform www.data.gov was officially launched, becoming the world’s first one-stop government data open platform. At present, more than 130,000 data sets have been opened for free download by the society, covering agriculture, more than 10 topics, such as business, climate, consumption, ecology, education, energy, finance, health, manufacturing, ocean, public safety, scientific research, and local government. The British government has also opened the Data.gov.uk website to open the data of the central government and local government departments to society. At present, dozens of countries and regions and international organizations (such as the OECD, the World Bank, etc.) are promoting open government data.

## Conclusion

The Chinese government attaches great importance to open government data. Since 2014, when open data policy was first written into the government work report by the State Council, China has successively issued a series of policy documents to promote open data projects, which are used to supervise officers, prevent corruption, and improve the quality of government services. The first open data project of local government was the service network of the Shanghai municipal government^2^ in June 2012. This project has covered 11 key areas of open data, including economic construction, resources and environment, education and technology, road transportation, social development, public safety, culture and leisure, health, people’s livelihood services, institutions and groups, and urban construction. Many other local governments are also actively building open data projects, such as Beijing, Hubei, Zhejiang, Guangdong, Tianjin, and Jiangsu.

The research findings of this article provide some insight into the impact of provincial governments’ authenticity and improve the level of open data and suggest the following: First, to further improve the level of open data of provincial governments; therefore, strengthening cooperation between government departments and disciplinary inspection departments. The results show that the PE, OPA, TLE, and ODU all affect the government’s integrity and reliability, so open government data should be vigorously promoted. In terms of policy availability guarantees, provincial governments have developed and maintained open data policies, standardized the data and its resources and management systems, improved data sharing organization and enforcement systems, and set up disciplinary inspection and monitoring departments. It can strengthen the cooperation in terms of building platforms of provincial governments. Government should coordinate the data disclosure resources of various departments, expand the scope of opening platforms, and ensure that disciplinary inspection and monitoring departments in the fight against corruption with the help of big data. At the technical stage, provincial departments should standardize the data entry procedures and standards, and provide key open data such as the use of funds for overseas trips, official disclosure information, real estate information, and other key information. The establishment of a database for open data should be expedited and constantly improve the list of project contractors purchased by the government agencies and the method of data sharing. In the case of usage and change, provincial governments should strengthen cooperation with disciplinary inspection and monitoring departments, including the data governance model of “Data Collection—Model Comparison—Verification—Feedback and Corrections.” Government should adapt and improve the functions of inquiry and decision.

Second, the level of government organization, as an important conditional variable that affects the effect of government integrity, needs to be constantly improved. Governments at the provincial level should adopt the “three lists” as a starting point to curb the power, reduce the abuse of power, such as approval, licensing, and procurement, and strengthen government internal control and oversight. Strengthen the government’s external oversight through key data management, monitoring public opinion, online monitoring and petition the monitoring, and accelerate the process of building a clean government.

Third, as one of the environmental barriers, “provincial pressure” also plays a direct role in the influencing of government reliability. Therefore, “provincial pressure” must be used to create a situation of systematic competition of local government opening figures within the province. We found that “the greater the pressure within the province,” the better the effect of government performance. It is therefore important to formulate relevant policies to guide different cities within the province to compare open data construction and performance, to establish a benchmark for data openness, and through effective competition, improve government integrity, transparency, and openness.

The study still has some limitations, mainly due to the impact on the integrity of the provincial government and the small size of the data openness level, and analysis only covers 3 years from 2019 to 2021. Four secondary indicators of the level of open data of provincial governments are from China’s Local Government Data Opening Report, and the data authority and its comprehensiveness still need to be improved.

With the development of the “14th 5-Year-Plan” and the acceleration in building digital government, the level of future local government data could be more comprehensively and systematically analyzed. Furthermore, this article focuses only on the analysis of the correlation between the impact of openness of provincial governments and the level of open data, but does not go into depth at the city level nor does it provide inter-provincial comparative analysis except for corruption registration cases.

## Data availability statement

The original contributions presented in the study are included in the article/supplementary material; further inquiries can be directed to the corresponding author.

## Author contributions

DX, MA, and XL contributed to the conception and design of the study. WQ and GX organized the database. MA performed the statistical analysis. WQ wrote the first draft of the manuscript. DX, MA, WQ, and GX wrote sections of the manuscript. All authors contributed to the article and approved the submitted version.

## Conflict of interest

The authors declare that the research was conducted in the absence of any commercial or financial relationships that could be construed as a potential conflict of interest.

## Publisher’s note

All claims expressed in this article are solely those of the authors and do not necessarily represent those of their affiliated organizations, or those of the publisher, the editors and the reviewers. Any product that may be evaluated in this article, or claim that may be made by its manufacturer, is not guaranteed or endorsed by the publisher.

## Footnotes

^1^Technological Level (TL) is a measurement of technological capabilities and sophistications.

^2^ https://data.sh.gov.cn/
